# Clinical value of AKR1B10 in hepatocellular carcinoma: A systematic review and meta-analysis

**DOI:** 10.1371/journal.pone.0279591

**Published:** 2022-12-30

**Authors:** Zixiang Wang, Yinxuan Pei, Weiwei Li, Jingxiao Zhang, Jinlong Liu

**Affiliations:** 1 Department of Hepatobiliary Surgery, Affiliated Hospital of Chengde Medical University, Chengde, Hebei Province, China; 2 Department of Pediatrics, Affiliated Hospital of Chengde Medical University, Chengde, Hebei Province, China; Tanta University Faculty of Medicine, EGYPT

## Abstract

**Background:**

To evaluate the clinical value of Aldo-keto reductase family 1 member B10 (AKR1B10) in the diagnosis and prognosis of hepatocellular carcinoma (HCC).

**Methods:**

A search of the PubMed, China Biology Medicine, Cochrane, and Embase databases was performed to conduct meta-analyses to evaluate the accuracy of AKR1B10 in diagnosing HCC and to assess the impact on prognosis of patients after curative resection of HCC.

**Results:**

A total of 12 different cohorts from 11 studies including 2747 HCC patients and 2053 controls showed that the pooled specificity and the pooled sensitivity of AKR1B10 for the diagnosis of HCC were 0.78 (95% CI: 0.69–0.85) and 0.85 (95% CI: 0.77–0.90), respectively. The pooled sensitivity and specificity of serum AKR1B10 for the diagnosis of HCC were 0.80 (95% CI: 0.70–0.86) and 0.87 (95% CI: 0.77–0.93), respectively. The pooled sensitivity and specificity of AKR1B10 in malignant tumor tissue for the diagnosis of HCC were 0.78 (95% CI: 0.61–0.89) and 0.82 (95% CI: 0.69–0.90), respectively. The pooled sensitivity and specificity of AKR1B10 to distinguish HCC from benign liver disease were 0.71 (95% CI: 0.62–0.78) and 0.84 (95% CI: 0.77–0.89), respectively. The sensitivity and specificity of AKR1B10 combined with alpha fetoprotein (AFP) in the diagnosis of HCC were 0.84 (95% CI: 0.79–0.88) and 0.88 (95% CI: 0.73–0.95), respectively. The pooled sensitivity and specificity of AKR1B10 in malignant tumor tissue for the diagnosis of early-stage HCC were 0.85 (95% CI: 0.62–0.95) and 0.88 (95% CI: 0.81–0.93), respectively. A meta-analysis of five studies including 798 patients demonstrated that high AKR1B10 expression in liver malignant tumor was associated with better overall survival in patients with HCC after hepatectomy (HR = 0.54, 95% CI: 0.41–0.72, p < 0.001).

**Conclusions:**

AKR1B10 exhibits a great clinical value in the diagnosis of HCC, especially for early-stage HCC, with excellent diagnostic accuracy. Furthermore, AKR1B10 expression can predict the prognosis of HCC patients after hepatic resection.

## Introduction

Hepatocellular carcinoma (HCC) is one of the most common malignant tumors worldwide [1–3]. Since most tumors are already in the advanced stage at the time of discovery, the 5-year overall survival (OS) rate of HCC is only 5–9% [4, 5]. However, it has been reported that the 5-year survival rate of patients can be improved to more than 50% if HCC can be diagnosed at an early stage [6]. The alpha-fetoprotein (AFP) is a marker for routine screening of HCC, but it is not stable for the detection of HCC. It has been reported that the sensitivity of AFP for diagnosing HCC ranges 63%–68% and the specificity ranges 76%–94% [7, 8]. Furthermore, AFP cannot satisfy the diagnosis of early HCC, Galle et al. found that 31% of early HCC patients had serum AFP levels below 20 ng / ml [9]. AFP expression levels were also found to be upregulated in patients with benign liver diseases, such as cirrhosis and hepatitis [10]. The limited diagnostic value of APF for HCC makes it urgent to find an excellent diagnostic biomarker for HCC. Currently, few studies have been performed on the predictive biomarkers after treatment of patients with HCC [9]. Therefore, many researchers have focused on identifying better diagnostic markers and prognostic markers for HCC.

Aldo-keto reductase family 1 member B10 (AKR1B10), originally identified as a human small intestinal aldose reductase and aldose reductase-like protein, is a cytosolic NADPH-dependent reductase that catabolizes a variety of endogenous compounds, such as dicarbonyls, aromatic, aliphatic aldehydes, and certain pharmaceutical ketones. The AKR1B10 gene is located on chromosome 7q33, and the AKR1B10 protein consists of 316 amino acids [11]. Recently, the enzyme was found to be highly expressed in several malignant tumors, including liver and lung tumors [12, 13]. As a result, AKR1B10 has attracted extensive attention as a relevant biomarker for the development of these tumors. In recent years, the rapid development of molecular testing technology has made it possible to detect AKR1B10 in peripheral blood samples, which allows analysis and provides clinicians with information on tumor biology. However, the diagnostic and prognostic significance of AKR1B10 in HCC remains unclear.

In the current study, we aimed to evaluate the diagnostic value of AKR1B10 in serum and tumor tissue for HCC, its ability to distinguish HCC from benign liver disease, and its accuracy in diagnosing early-stage HCC. Furthermore, we aimed to explore the impact of AKR1B10 expression on the OS and recurrence-free survival (RFS) of HCC patients after hepatectomy.

## Methods

### Data sources and searches

Literature search was performed on PubMed, China Biology Medicine, Cochrane, and Embase databases to identify studies reporting the diagnostic and prognostic value of AKR1B10 in patients with HCC. The deadline for retrieval was April 2, 2022. The search terms included in the search strategy were as follows: ((AKR1B10) OR (Aldo-keto reductase1-B10)) AND ((liver cancer) OR (hepatoma) OR (hepatic carcinoma) OR (hepatocellular carcinoma)).

### Screening and selection of studies

All studies were individually screened by the title and abstract, followed by a full-text assessment by two reviewers. Divergences were solved by discussion until consensus was reached. The inclusion criteria were as follows: (1) research population included patients with HCC and non-HCC (benign liver disease or normal person); (2) AKR1B10 was analyzed in all patient groups; (3) prognostic studies needed to provide sufficient data to calculate the HR and its 95% CI; (4) diagnostic studies needed to provide sufficient data to calculate sensitivity and specificity; and (5) there were no language restrictions. The exclusion criteria were as follows: (1) insufficient data provided to calculate the HR; (2) insufficient data provided to calculate the sensitivity and specificity; (3) abstracts, reviews, letters, case reports, or publications; or (4) animal or cell experiments.

### Data extraction and outcomes

The data were individually extracted by two authors, following a predefined scheme. Divergences were solved by discussion until consensus was reached. Extracted study characteristics included the following: first author, publication year, type of cancer, number of patients, number of controls, type of sampling, aim of the study, and detection method of the AKR1B10 expression level. On the basis of the aim and outcome of the included studies, studies were classified into the diagnostic and prognostic groups. Studies were classified into the diagnostic group when AKR1B10 was used as a biomarker to diagnose HCC or to differentiate HCC from healthy controls. Studies were categorized into the prognostic group when the association between the high or low expression level of AKR1B10 and survival was provided.

### Study quality assessment

The quality of included studies was scored separately by two reviewers. Divergences were solved by discussion until consensus was reached. The quality assessment of diagnostic accuracy studies (QUADAS) criteria was used to assess the quality of studies related to the diagnosis of HCC by AKR1B10. The Newcastle Ottawa scale was used to assess the quality of studies related to the prognosis of HCC.

### Statistical analysis

All analyses were conducted using Stata 16 and Meta-Disc 1.4 software. For diagnostic studies, the pooled sensitivity, specificity, positive likelihood ratio (PLR), negative likelihood ratio (NLR), diagnostic odds ratio (DOR), and area under the curve (AUC) and their 95% confidence intervals (CIs) were calculated to assess the diagnostic value of AKR1B10 for HCC. Furthermore, the Spearman correlation analysis method was used to examine threshold effect heterogeneity among diagnostic studies. For prognostic studies, the hazard ratio (HR) and its 95% CI were collected to assess the correlation between AKR1B10 expression and prognosis of HCC. If the study only provided Kaplan Meier survival curves, the Engage Digitizer version 2.11 software was used to extract the relevant values from the survival curves and calculate the HR (95% CI) [14, 15]. The test of inconsistency (I^2^) was used to evaluate the degree of inter-study statistical heterogeneity. Substantial heterogeneity was indicated if I^2^ values were ≥ 50%, and the random-effects model was adopted [16, 17]. The Deeks’ funnel plot asymmetry test was used to assess the publication bias of the included studies, and P < 0.05 was considered statistically significant [18].

## Results

### Characteristics of the included studies

**Fig 1:**
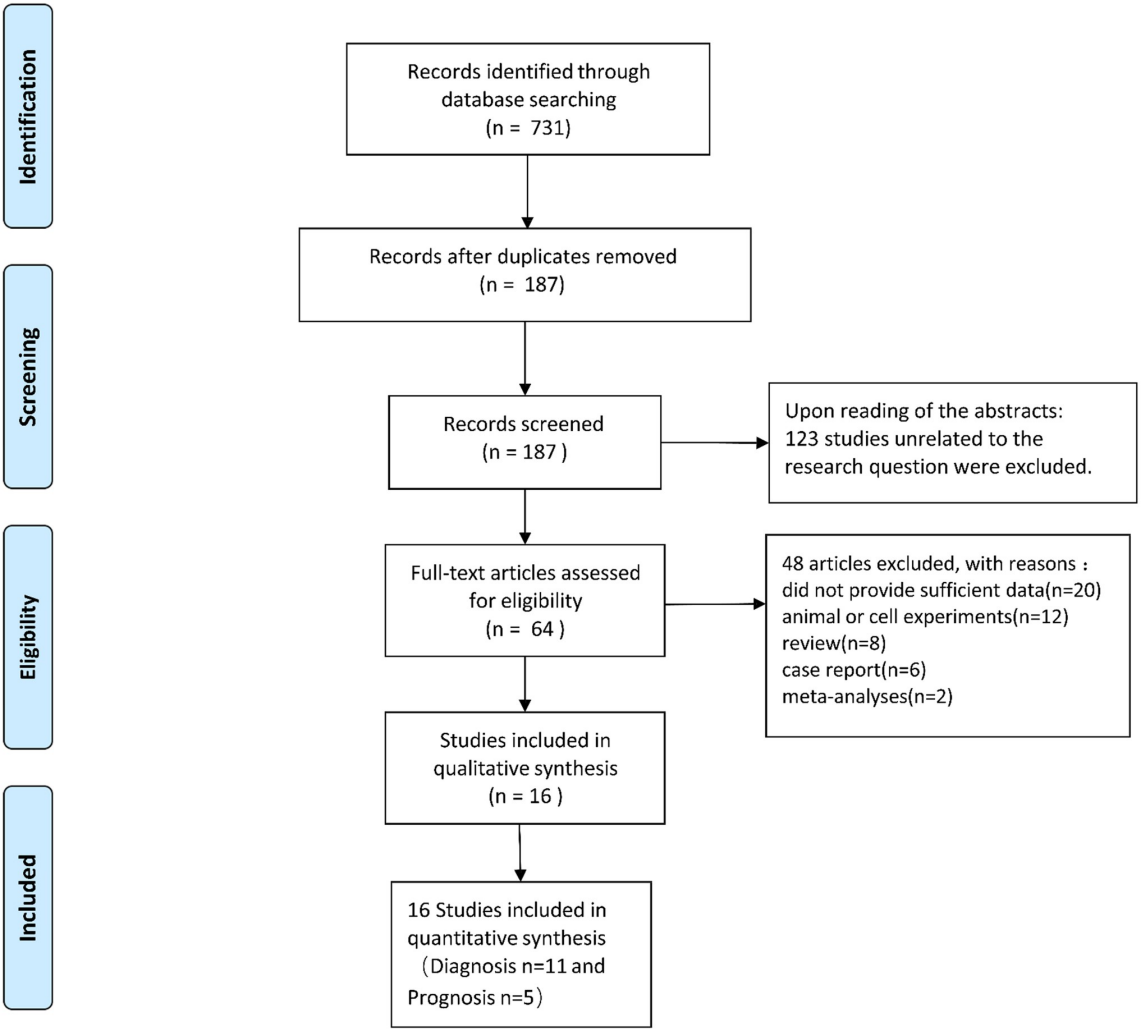
The flow diagram of study selection.

The flowsheet of the study screening process is shown in Fig 1. Database searches yielded 731 studies. A total of 187 studies were found after removing duplicates. Upon reading the abstracts, 123 studies unrelated to the research question were excluded. The remaining 64 articles were fully reviewed, and 48 articles were removed (20 articles did not provide sufficient data, eight were reviews, six were case reports, two were meta-analyses, and 12 were animal or cell experiments). Overall, 16 studies, including 11 diagnostic [19–29] and five prognostic [30–34] studies, were included in our meta-analysis.

#### Analysis of threshold effect

To verify whether there was heterogeneity caused by the AKR1B10 threshold among the included diagnostic studies, we used the Meta-Disc 1.4 software to perform the Spearman test to detect the threshold effect, and the correlation coefficient and P value between the sensitivity logit and the 1—specificity logit were calculated. The results showed that the Spearman correlation coefficient and P value were -0.413 and 0.183 (> 0.05), respectively, indicating that the heterogeneity caused by different AKR1B10 thresholds did not exist in the 11 diagnostic studies.

### Accuracy of AKR1B10 for diagnosing HCC

**Fig 2:**
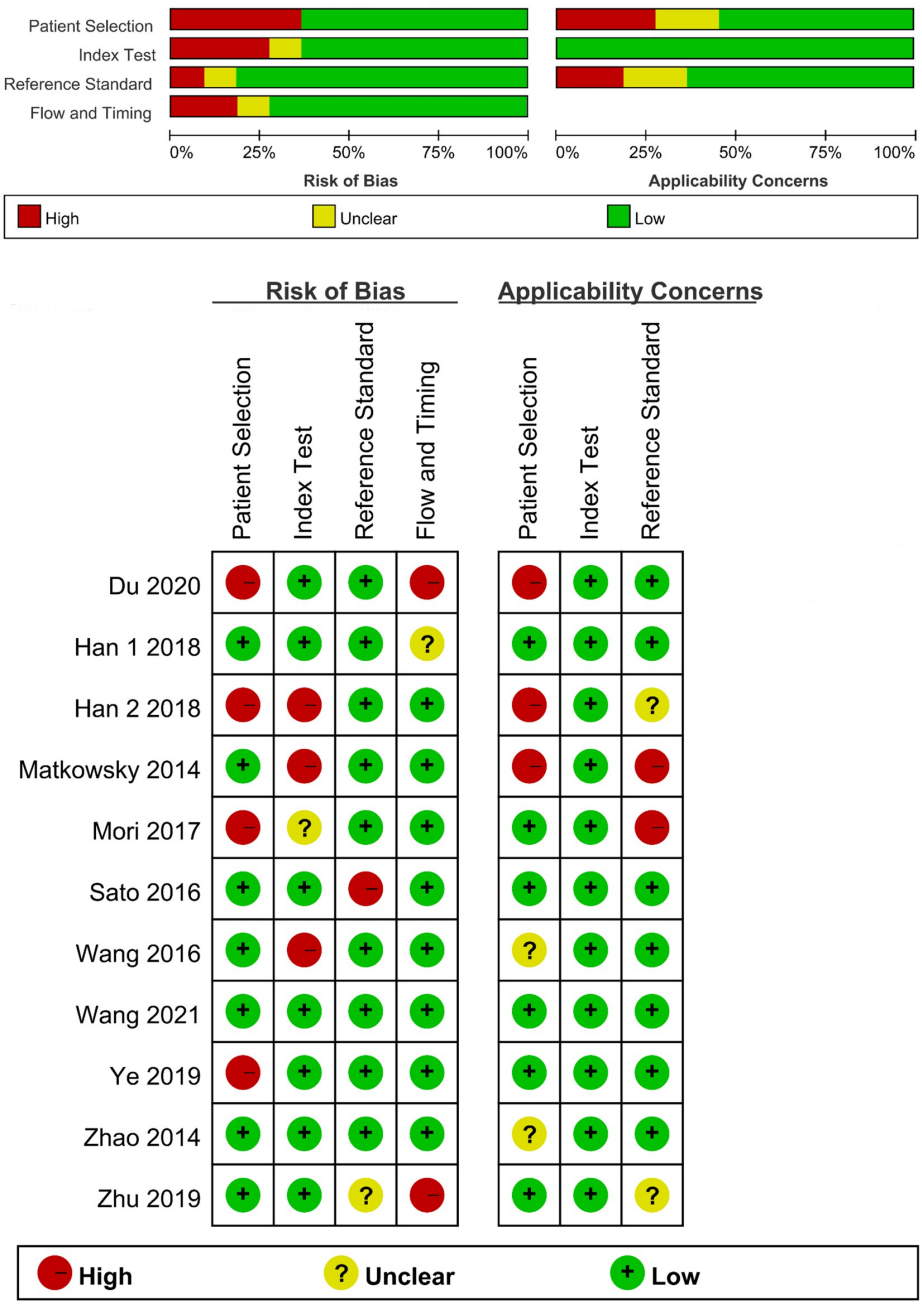
Overall methodology quality assessment of the included studies using the QUADAS criteria.

A total of 11 studies [19–29] including 12 different cohorts reported data evaluating the diagnostic accuracy of AKR1B10 for HCC, and they were published between 2014 and 2020. Data from 2747 HCC patients and 2053 controls were collected. All diagnoses of HCC patients were verified by histopathology. Histopathology, ultrasound (US), computed tomography (CT), magnetic resonance imaging (MRI) or hematology were used to diagnose all patients with benign liver disease. The Barcelona Clinic Liver Cancer (BCLC) and tumor node metastasis (TNM) classifications were used to define HCC stages in the included studies. Enzyme linked immunosorbent assay (ELISA), Time-resolved fluoroimmunoassay (TRFIA), Reverse transcription polymerase chain reaction (PT-PCR) and Immunohistochemistry was used to determine the expression level of AKR1B10 in serum and tumor tissue. Detailed characteristics of the included studies are shown in Table 1. Fig 2 shows the details of the quality assessment form.

**Table 1:** Characteristics of the studies included in this meta-analysis.

Author	Year	Country	HCC group		HCC Stage	Controls group		Detection method	Sample type	Cut off
			Population	Diagnostic criteria		Population	Diagnostic criteria			
Han 1 [19]	2018	China	84 HCC	Histopathology	38/46	74 LC, 79 CH	Hematology	ELISA	serum	SAL 1510 pg/ml
Du [20]	2020	China	16 HCC	Histopathology	5/11	24 HC, 24 LC	Histopathology US, CT, MRI	ELISA	serum	SAL 122290 pg/mL
Zhu [21]	2019	China	170 HCC	Histopathology	45/125*	120 HC	NA	TRFIA	serum	SAL 232.7 pg/ml
Wang [22]	2020	China	160 HCC	Histopathology	76/84	120 HC, 160 LC,	Hematology	ELISA	serum	SAL 903.0 pg/ml
						120 CHB,112 OLD				
Ye 1 [23]	2019	China	209 HCC	Histopathology	79/130	203 HC, 40 LC,	US, CT, MRI	ELISA	serum	SAL 267.9 pg/ml
						10 CHB, 57 BLD				
Ye 2 [23]	2019	China	204 HCC	Histopathology	75/129	208 HC, 38 LC,	US, CT, MRI	ELISA	serum	SAL 267.9 pg/ml
						22 CHB, 50 BLD				
Matkowsky [24]	2014	America	89 HCC	Histopathology	84/5*	24 HA, 9 FNH	Histopathology	Immunohistochemistry	tissue	PCER 5%
Mori [25]	2017	Japan	13 HCC	Histopathology	NA	106 HBV	US, CT, MRI	RT-PCR	tissue	PCER 15%
Han 2 [26]	2018	China	44 HCC	Histopathology	29/15	37 LC	Histopathology	Immunohistochemistry	tissue	IOD≥89.5
Sato [27]	2016	Japan	55 HCC	Histopathology	NA	507 HBV	Hematology	RT-PCR	tissue	PCER 6%
							US, CT, MRI			
Zhao [28]	2014	China	89 HCC	Histopathology	NA	26 BLD	US, CT, MRI	Immunohistochemistry	tissue	NA
Wang [29]	2016	China	106 HCC	Histopathology	NA	10 HC, 106 PT	Histopathology	RT-PCR	tissue	NA
Sonohara [30]	2016	Japan	132 HCC	Histopathology	98/58*	26 HCC	Histopathology	RT-PCR	tissue	NA
Schmitz [31]	2011	Germany	81 HCC	Histopathology	NA	87 HCC	Histopathology	Immunohistochemistry	tissue	NA
Wang [32]	2017	China	67 HCC	Histopathology	67/43	43 HCC	Histopathology	Immunohistochemistry	tissue	NA
Ha [33]	2014	Korea	125 HCC	Histopathology	138/117	130 HCC	Histopathology	Immunohistochemistry	tissue	NA
Li [34]	2018	China	59 HCC	Histopathology	NA	39 HCC	Histopathology	Immunohistochemistry	tissue	NA

LC: Liver cirrhosis; CH: Chronic hepatitis; HC: Healthy control; HBV: hepatitis B virus; PVT: Portal vein thrombosis; CHB: Chronic hepatitis B; OLD: Other liver diseases; BLD: Benign liver disease; HA: Hepatic adenoma; FNH: Focal nodular hyperplasia; PT: Paracancerous tissues; *: indicates the number of HCC patients with TNM stage I and II/ TNM stage III and IV, No * in this column indicates the number of HCC patients with BCLC phase 0 and A HCC / BCLC phase B, C and D; NA = not available; ELISA: Enzyme linked immunosorbent assay; TRFIA: Time-resolved fluoroimmunoassay; RT-PCR: Reverse transcription polymerase chain reaction; SAL: serum AKR1B10 level; PCER: Positive cell expression ratio; IOD: integral optical density.

**Fig 3:**
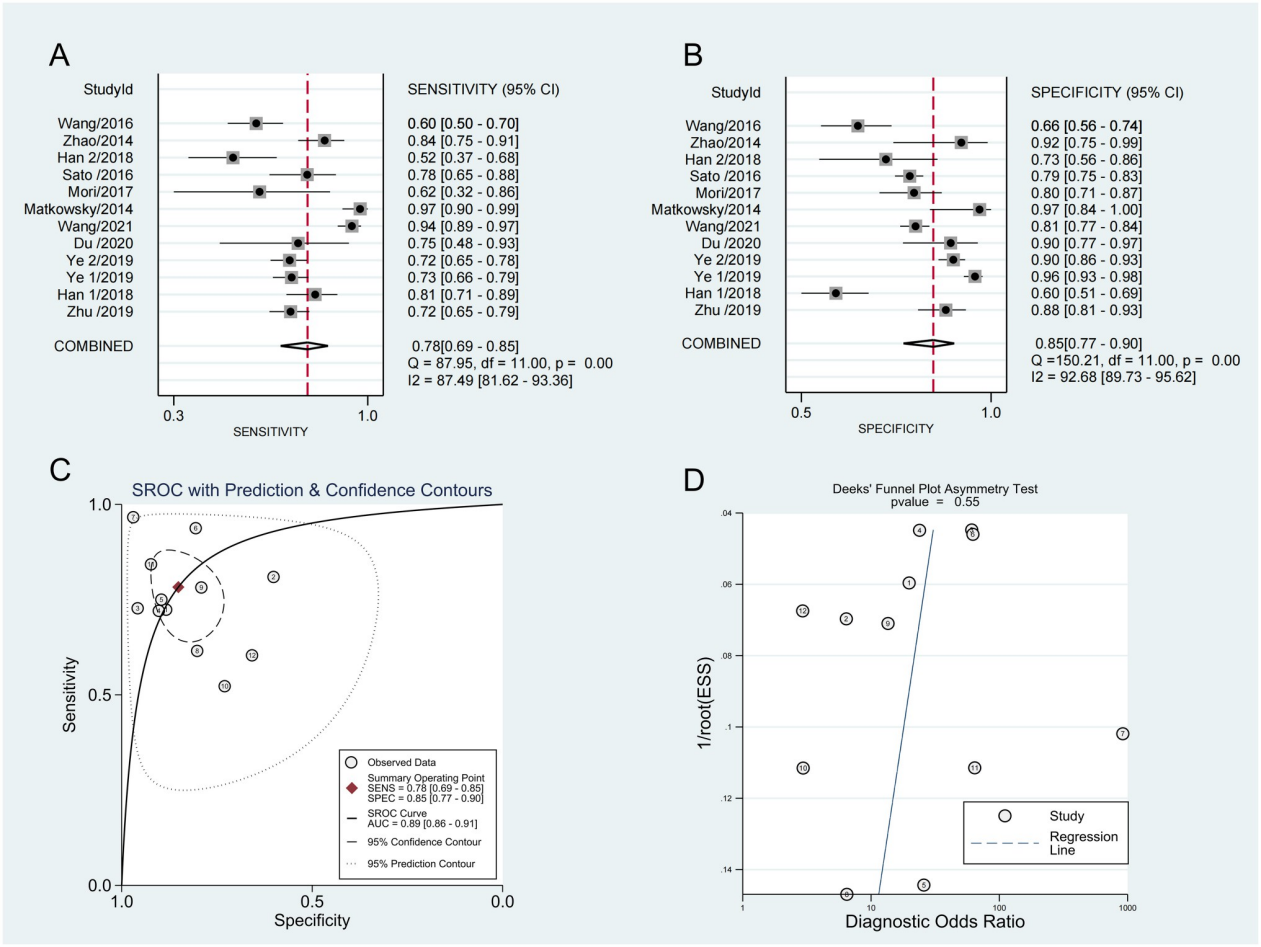
Forest plots of sensitivity (A), specificity (B), area under the curve (AUC) (C), and funnel plot (D) of AKR1B10 for diagnosing HCC among 11 studies.

The pooled sensitivity and specificity of AKR1B10 in discriminating HCC from non-HCC were 0.78 (95% CI: 0.69–0.85) and 0.85 (95% CI: 0.77–0.90), respectively. The PLR, NLR, DOR, and AUC with their corresponding 95% CIs were 5.3 (95% CI: 3.3–8.5), 0.26 (95% CI: 0.17–0.38), 21.00 (95% CI: 9.00–46.00), and 0.89 (95% CI: 0.86–0.91), respectively (Fig 3A–3C). Our meta-analysis suggested that AKR1B10 has good diagnostic efficacy for HCC. However, we found that each article had a different source of AKR1B10. In addition, benign liver disease was used as a control group in five studies [23, 25–28]. Therefore, we performed subgroup analyses to further evaluate the accuracy of AKR1B10 as a diagnostic marker for HCC.

#### Accuracy of AKR1B10 derived from different samples and detection methods for the diagnosis of HCC

The tested samples of AKR1B10 included serum and malignant tumor tissue. To verify whether different sources of AKR1B10 have different diagnostic accuracies for HCC, we analyzed the diagnostic accuracy of AKR1B10 levels for HCC in two samples. A total of five articles [19–23] including 6 different cohorts reported the diagnostic accuracy of AKR1B10 in serum for HCC. As shown in Table 2, the pooled sensitivity and specificity of serum AKR1B10 for the diagnosis of HCC were 0.80 (95% CI: 0.70–0.86) and 0.87 (95% CI: 0.77–0.93), respectively. The PLR, NLR, DOR, and AUC with their corresponding 95% CIs were 6.1 (95% CI: 3.5–10.7), 0.24 (95% CI: 0.16–0.34), 26.00 (95% CI: 14.00–49.00), and 0.89 (95% CI: 0.86–0.92), respectively. In addition, a total of six studies [24–29] reported the diagnostic accuracy of AKR1B10 in the malignant tumor tissue for HCC. The pooled sensitivity and specificity of AKR1B10 in malignant tumor tissues for the diagnosis of HCC were 0.78 (95% CI: 0.61–0.89) and 0.82 (95% CI: 0.69–0.90), respectively. The PLR, NLR, DOR, and AUC with their corresponding 95% CIs were 4.3 (95% CI: 2.1–8.9), 0.27 (95% CI: 0.13–0.57), 16.00 (95% CI: 4.00–69.00), and 0.87 (95% CI: 0.84–0.90), respectively (Table 2). Pooled analysis showed that AKR1B10 in the malignant tumor tissue had lower pooled sensitivity, specificity, and AUC for diagnosing HCC compared with AKR1B10 in serum.

**Table 2:** Summary of subgroup analyses.

	Serum	malignant tumor tissue	HCC VS BLD	AKR1B10+AFP	Early HCC
SEN	0.80(95% CI: 0.70–0.86)	0.78 (95% CI: 0.61–0.89)	0.71(95% CI: 0.62–0.78)	0.84(95% CI: 0.79–0.88)	0.85(95% CI: 0.62–0.95)
SPE	0.87(95% CI: 0.77–0.93)	0.82 (95% CI: 0.69–0.90)	0.84(95% CI: 0.77–0.89)	0.88(95% CI: 0.73–0.95)	0.88(95% CI: 0.81–0.93)
PLR	6.1(95% CI: 3.5–10.7)	4.3(95% CI: 2.1–8.9)	4.3(95% CI: 2.9–5.6)	7.1(95% CI: 3.0–16.8)	7.3(95% CI: 4.3–12.4)
NLR	0.24(95% CI: 0.16–0.34)	0.27(95% CI: 0.13–0.57)	0.35(95% CI: 0.25–0.47)	0.18 (95% CI: 0.14–0.22)	0.17(95% CI: 0.06–0.49)
DOR	26.00(95% CI: 14.00–49.00)	16.00(95% CI: 4.00–69.00)	13.00(95% CI: 6.00–25.00)	40.00(95% CI: 18.00–86.00)	42.00(95% CI: 11.00–160.00)
AUC	0.89(95% CI: 0.86–0.92)	0:87(95% CI: 0.84–0.90)	0.85(95% CI: 0.82–0.88)	0.90(95% CI: 0.87–0.92)	0.92(95% CI: 0.90–0.94)

BLD: Benign liver diseases; SEN: Sensitivity; SPE: Specificity; PLR: Positive likelihood ratio; NLR: Negative likelihood ratio; DOR: diagnostic odds ratio; AUC: area under the curve.

**Fig 4:**
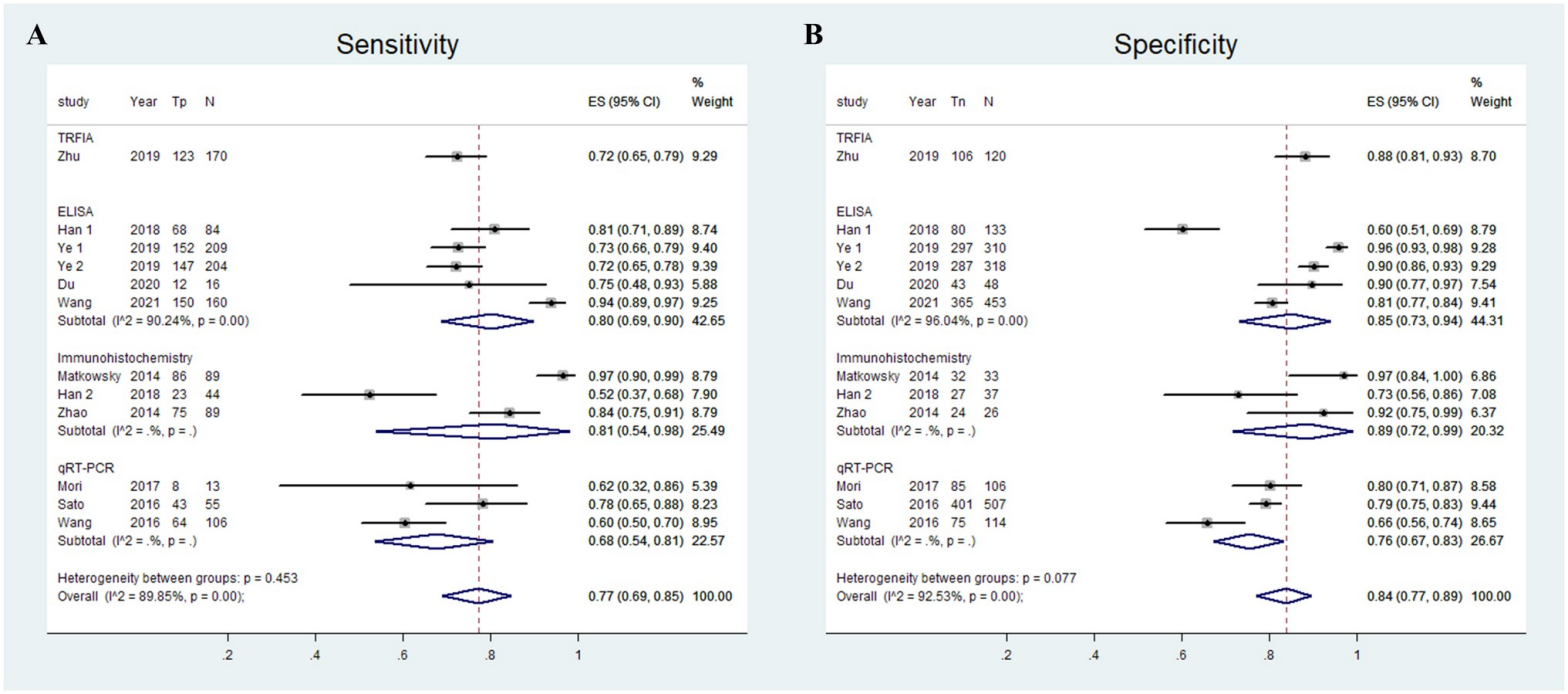
Pooled sensitivity and specificity of AKR1B10 levels measured by different detection methods for the diagnosis of HCC.

To verify whether AKR1B10 levels detected by different detection method have different diagnostic accuracy for HCC, a subgroup analysis was performed. ELISA was used to measure AKR1B10 expression levels in serum in four studies [19, 20, 22, 23], and the pooled sensitivity and specificity for diagnosing HCC using AKR1B10 expression levels measured by ELISA were 0.80 (95% CI: 0.69–0.90) and 0.85 (95% CI: 0.73–0.94), respectively (Fig 4). Three studies [24, 26, 28] used Immunohistochemistry as detection method of AKR1B10 to diagnose HCC, and the pooled sensitivity and specificity were 0.81 (95% CI: 0.54–0.98) and 0.89 (95% CI: 0.72–0.99), respectively (Fig 4). RT-PCR was used to measure AKR1B10 expression levels to diagnose HCC in three studies [25, 27, 29], and the pooled sensitivity and specificity were 0.68 (95% CI: 0.54–0.81) and 0.76 (95% CI: 0.67–0.83), respectively (Fig 4).

### Accuracy of AKR1B10 in distinguishing HCC from benign liver diseases

To assess the accuracy of AKR1B10 in distinguishing HCC from benign liver diseases, such as chronic hepatitis and cirrhosis, we collected and analyzed the relevant data from five studies [23, 25–28] including six different cohorts. The pooled sensitivity and specificity of AKR1B10 in distinguishing HCC from benign liver disease were 0.71 (95% CI: 0.62–0.78) and 0.84 (95% CI: 0.77–0.89), respectively. The PLR, NLR, DOR, and AUC with their corresponding 95% CIs were 4.3 (95% CI: 2.9–5.6), 0.35 (95% CI: 0.25–0.47), 13.00 (95% CI: 6.00–25.00), and 0.85 (95% CI: 0.82–0.88), respectively (Table 2). Therefore, we conclude that AKR1B10 has good ability to distinguish HCC from benign liver disease.

### Accuracy of AKR1B10 combined with AFP for the diagnosis of HCC

AFP is a traditional diagnostic marker for HCC. To evaluate the diagnostic accuracy of AKR1B10 combined with AFP for HCC, a total of five articles [19–23] including six different cohorts were collected and analyzed. Patients could be diagnosed with HCC if they were either AFP positive or AKR1B10 positive. The pooled sensitivity and specificity of AKR1B10 combined with AFP in discriminating HCC from non-HCC were 0.84 (95% CI: 0.79–0.88) and 0.88 (95% CI: 0.73–0.95), respectively. The PLR, NLR, DOR, and AUC with their corresponding 95% CIs were 7.1 (95% CI: 3.0–16.8), 0.18 (95% CI: 0.14–0.22), 40.00 (95% CI: 18.00–86.00), and 0.90 (95% CI: 0.87–0.92), respectively (Table 2).

### Accuracy of AKR1B10 in the diagnosis of early HCC

A total of three studies [22–24] including four different cohorts provided relevant data for the diagnosis of early HCC by AKR1B10. HCC at stages 0 and A was defined as early HCC when the BCLC classification was used [22, 23]. When the TNM classification was used [24], HCC at stages I-II was defined as early HCC. The sensitivity and specificity of AKR1B10 for diagnosing early-stage HCC were 0.85 (95% CI: 0.62–0.95) and 0.88 (95% CI: 0.81–0.93), respectively. The PLR, NLR, DOR, and AUC with their corresponding 95% CIs were 7.3 (95% CI: 4.3–12.4), 0.17 (95% CI: 0.06–0.49), 42.00 (95% CI: 11.00–160.00), and 0.92 (95% CI: 0.90–0.94), respectively (Table 2). Our meta-analysis suggests that AKR1B10 has an excellent diagnostic efficacy for early HCC.

### Clinical value of AKR1B10 for the prognosis of HCC

**Fig 5:**
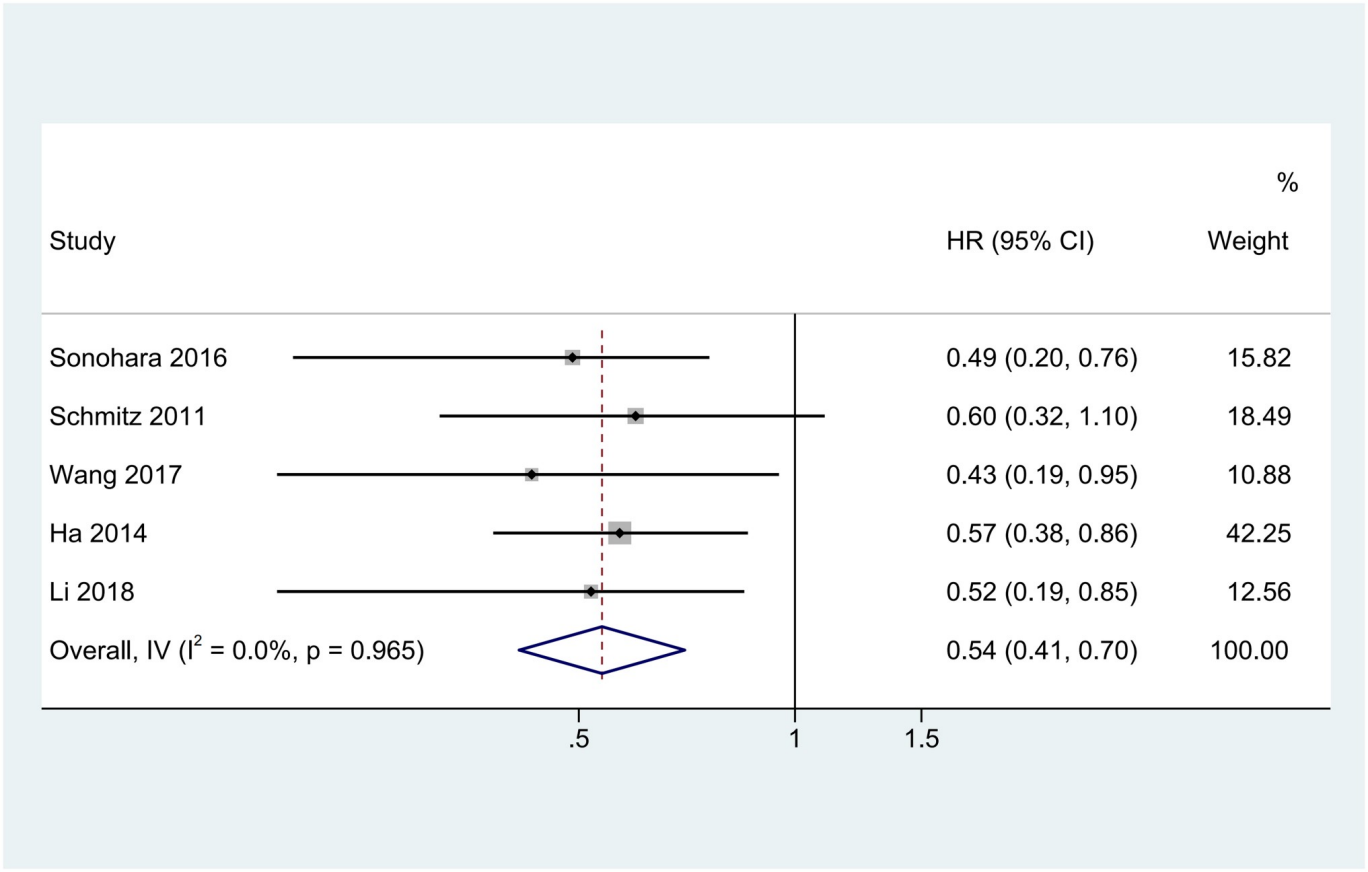
Forest plot of the HR for the association between high AKR1B10 expression and overall survival in patients after hepatectomy.

A total of five studies [30–34] including 798 patients reported the data on the ability of the AKR1B10 expression level to predict the OS of patients after hepatectomy. All HCC patients included in the study had undergone hepatectomy, and AKR1B10 expression levels in tissues of liver malignancy were measured by Immunohistochemistry or RT-PCR. HCC patients with high expression of AKR1B10 were divided into the HCC group and HCC patients with low expression were divided into the control group. We extracted the hazard ratio (HR) and standard error for OS from each study. The HR in Schmitz’s study [31] was derived from the K-M curve, other HRs were obtained directly from the corresponding original articles. If HR was < 1, it indicated that the OS of patients after hepatectomy with AKR1B10 high expression was better than that after hepatectomy with AKR1B10 low expression. As shown in Fig 5, our study suggests that high AKR1B10 expression in malignant tumor tissue was associated with better OS in patients with HCC after hepatectomy (HR = 0.54, 95% CI: 0.41–0.72, p < 0.001) In addition, Sonohara [30] and Ha [33] showed that high AKR1B10 levels are associated with better RFS in HCC patients after hepatic resection. These results suggest that high AKR1B10 expression may be a marker of good prognosis in HCC patients after hepatic resection.

### Publication bias

To assess the publication bias of included studies, the linear regression test of Deeks’ funnel plot asymmetry was conducted. The pooled Deeks’ test result of the diagnostic studies was p = 0.55 (Fig 3D), which indicated that there was no obvious publication bias for our diagnostic analysis.

## Discussion

In HCC cells, inhibition of AKR1B10 expression resulted in cell cycle arrest and impaired cell proliferation, suggesting that AKR1B10 may exert tumorigenic effects through enhanced cell growth [35]. Upregulated AKR1B10 expression levels have been proven to be a risk factor for HCC [25, 27]. Furthermore, experimental results in vitro and in vivo demonstrated that AKR1B10 is involved in cancer cell proliferation [36, 37]. Therefore, we had a reason to believe that AKR1B10 plays an important role in the course of HCC, and we performed meta-analyses to evaluate the clinical value of AKR1B10 in HCC.

In this meta-analysis, after excluding heterogeneity due to threshold effects, we showed that the overall sensitivity and specificity of AKR1B10 for the diagnosis of HCC were 78% and 85%, respectively. Subsequently, we analyzed the main sources of heterogeneity and divided the included diagnostic studies into six subgroups. Our study showed that the sensitivity and specificity of serum and tissue as test specimens for AKR1B10 in the diagnosis of HCC were similar, which indicates that the different tested specimens were not the main source of heterogeneity. In addition, the control population was not consistent among the included studies, which may have affected the accuracy of AKR1B10 for diagnosing HCC.

With the development of science and technology, simultaneous determination of AKR1B10 and AFP is feasible. Our meta-analysis table indicates a higher sensitivity and specificity of AKR1B10 combined with AFP for the diagnosis of HCC compared with AKR1B10 or AFP alone. In addition, serum AFP levels have been reported to be not only associated with HCC but also to be elevated in patients with benign liver diseases, such as hepatitis and cirrhosis [10, 38]. Our meta-analysis indicated that AKR1B10 as a tumor marker has the ability to distinguish HCC from benign liver disease.

It has been reported that the 5-year survival rate of patients can be improved to more than 50% if HCC can be detected at an early stage [6]. However, Marrero’s study [39] showed that the sensitivity and specificity of AFP for the diagnosis of early HCC were 66% and 82%, respectively. Our meta-analysis indicated that the sensitivity of AKR1B10 for the diagnosis of early-stage HCC is higher than that of AFP. This may be related to the opposite expression pattern of AKR1B10 compared with that of AFP [19]. It has been reported that the unique expression pattern of AKR1B10 may be because of 14-3-3 ε induced AKR1B10 upregulation at the early stage of HCC, leading to decreased retinoic acid levels and subsequent cell proliferation. In advanced HCC, the relationship between 14-3-3 ε and AKR1B10 is inhibited by unknown factors, leading to decreased AKR1B10 levels [40].

The main difficulty in treating HCC is the high frequency of tumor recurrence even after liver transplantation and radical resection [41], which cannot be avoided even in well-differentiated tumors [42]. In the present meta-analysis, summary results without significant heterogeneity showed that high expression of AKR1B10 in malignant tumor tissue was significantly correlated with better OS and RFS in HCC patients after hepatectomy. Liu’s study [43] suggested that this may be because of the complex regulation of expression at the time of AKR1B10 transcription. In the AKR1B10 promoter, multiple putative binding sites for oncogenic and tumor suppressor proteins are recognized, including p53, cets-1, and C/EBP. In more malignant HCC cells, the deregulation or nonspecific switching on/off of certain transcriptional machinery may downregulate AKR1B10 expression.

To the best of our knowledge, no meta-analysis has yet been reported on the association between AKR1B10 expression levels and the diagnosis and prognosis of HCC. However, our study has some limitations. First, patients from different included studies had different characteristics, which are common in the evaluation of the accuracy of diagnostic tests. It may have affected the accuracy of diagnostic analysis. Second, the cut-off for AKR1B10 positivity was not uniform in the literature included in this study, which may have affected the accuracy of AKR1B10 for HCC diagnosis. Finally, most of the studies on AKR1B10 for diagnosing HCC were conducted in Asian populations, which may have resulted in a population selection bias.

## Conclusions

In conclusion, our meta-analysis suggests that AKR1B10 has a high diagnostic value for HCC and can be used as a novel screening method. Notably, AKR1B10 has excellent diagnostic efficacy for early-stage HCC and shows good performance when combined with AFP to diagnose HCC. In addition, AKR1B10 can predict OS and RFS of HCC patients after hepatectomy. However, more large cohort studies or large randomized controlled multicenter trials are needed to further explore the clinical value of AKR1B10 for patients with HCC.

## Supporting information

S1 Checklist PRISMA 2020 checklist. (PDF)
